# Treatment of Mammary Abscess

**Published:** 1848

**Authors:** 


### ARTICLE XI.

### *On the Treatment of Mammary Abscess.*

The following is a case of no very unusual occurrence: A mother loses her infant, and broken-hearted at the event, she neglects to pay that attention to herself which her case requires. No attempts being made to relieve the distended breast of its secretion, the gland becomes hard and painful, and at length abscesses form in different situations. if the treatment is confined to the evacuation of matter by puncture, and to the application of fomentations and poultices but little good is done. The former abscesses continue to discharge; fresh ones are constantly forming, and the woman at length sinks in a state of hectic. Means should be used to stop the secretion of milk, which has been going on all this time, and perpetuating the mischief. We have no bettter means of affecting this than by the administration of a hydragogue purgative. I am usually in the habit of prescribing the sulphate of magnesia in the compound infusion of roses, to which, when there is much hectic and debility, I add some quinine, and dilute sulphuric acid. The effect of this treatment is sometimes almost magical. I have known a woman, who for months had been suffering from a succession of mammary abscess, begin to get well from the moment that the salts produced its liquid evacuations from the bowels; the secretion of milk ceased, and the purulent discharge diminished, a more adhesive inflammation being established in the place of these two actions. *Prov. Med. and Surg. Jour.* Braith's Ret.

